# GBNet: Deciphering regulatory rules in the co-regulated genes using a Gibbs sampler enhanced Bayesian network approach

**DOI:** 10.1186/1471-2105-9-395

**Published:** 2008-09-24

**Authors:** Li Shen, Jie Liu, Wei Wang

**Affiliations:** 1 Department of Chemistry and Biochemistry, University of California, San Diego, California, USA

## Abstract

**Background:**

Combinatorial regulation of transcription factors (TFs) is important in determining the complex gene expression patterns particularly in higher organisms. Deciphering regulatory rules between cooperative TFs is a critical step towards understanding the mechanisms of combinatorial regulation.

**Results:**

We present here a Bayesian network approach called GBNet to search for DNA motifs that may be cooperative in transcriptional regulation and the sequence constraints that these motifs may satisfy. We showed that GBNet outperformed the other available methods in the simulated and the yeast data. We also demonstrated the usefulness of GBNet on learning regulatory rules between YY1, a human TF, and its co-factors. Most of the rules learned by GBNet on YY1 and co-factors were supported by literature. In addition, a spacing constraint between YY1 and E2F was also supported by independent TF binding experiments.

**Conclusion:**

We thus conclude that GBNet is a useful tool for deciphering the "grammar" of transcriptional regulation.

## Background

Decoding regulatory interactions between transcription factors (TFs) and their target genes is critical in understanding the complex gene expression patterns in response to extra- or intra-cellular signals. Many computational methods have been developed to identify the cis-regulatory elements recognized by TFs [1]. These DNA motifs have also been determined by experimental measurements [2]. The accumulation of known TF motifs facilitates addressing a more challenging question, understanding the combinatorial regulation of TFs and deciphering the rules of how the TFs cooperate with each other, which is particularly important for studying transcriptional regulation in higher organisms [3].

The previous efforts have been mainly focused on inferring which TFs may function together [4-13]. However, these studies cannot reveal the regulatory mechanisms of combinatorial regulation, namely whether a TF motif has a positional preference relative to the transcription start site (TSS) or whether the order of the two motifs matters for their cooperation. The importance of such regulatory "grammar" has been observed in numerous studies. For example, the binding site of the repressor Giant relative to those of the Gal4 activators determined transcription of a reporter gene in the embryo of *Drosophila melanogaster *[14].

Searching for the sequence constraints between TF motifs is a difficult task. As we will see below, simple enumeration of all possible sequence features and conducting statistical test to evaluate the significance for each of them is computationally expensive for even a modest number of candidate motifs. Alternative methods are thus needed to tackle this problem. Recently, Elemento *et al*. developed a motif finding algorithm called FIRE that can predict various sequence constraints of motifs and whether pairs of motifs interact with each other [15]. FIRE is based on the mutual information between the sequence features of interest and gene expression. However, because Elemento *et al*. emphasized on removing false positives, the relative small number of predicted motifs implies that some true motifs might be missed. For example, only 17 DNA motifs and 6 RNA motifs were identified from 78 clusters generated from 173 microarray experiments in yeast [15]. In addition, FIRE only considers consensus sequences with mismatches, which is a relatively simple representation of motifs compared with position weight matrix. More importantly, FIRE cannot consider the joint effects of multiple rules. The rules were tested individually by FIRE and the computational cost would be too high to enumerate all possible combinations of rules (see below). Therefore, for example, synergy between rules cannot be detected by FIRE.

In the present study, we adopted a Bayesian network approach to identify regulatory grammars because Bayesian network explicitly models the nonlinear relationship between sequence rules. Our goal is to find enriched constraints for DNA motifs such as spacing between TF binding sites and positional bias of a TF sites relative to TSS in a group of sequences, often promoter sequences of a set of co-regulated genes. This can be considered as a generalization of motif finding algorithms. It is important to emphasize that we do not aim to predict gene expression based on sequences, which is the goal of the studies of, such as, Beer and Tavazoie [16] and Yuan et al. [17].

We implemented a Gibbs sampling procedure to search for optimal Bayesian network structure. We call our method GBNet, **G**ibbs sampler enhanced **B**ayesian **Net**work, and the software is available at . To demonstrate the strength of our searching strategy, we compared the performance of GBNet with BBNet, in which a greedy searching algorithm is implemented to search for the optimal Bayesian network. We have applied both methods to simulated data as well as yeast and human data. The results showed that Gibbs sampling has much better performance than greedy search in searching for sequence constraints between cooperative TFs. We also demonstrated that numerous sequence features identified by GBNet for human transcription factor YY1 were supported by literature and experimental evidence.

## Results

### GBNet: a Gibbs-sampler enhanced Bayesian network

**Figure 1:**
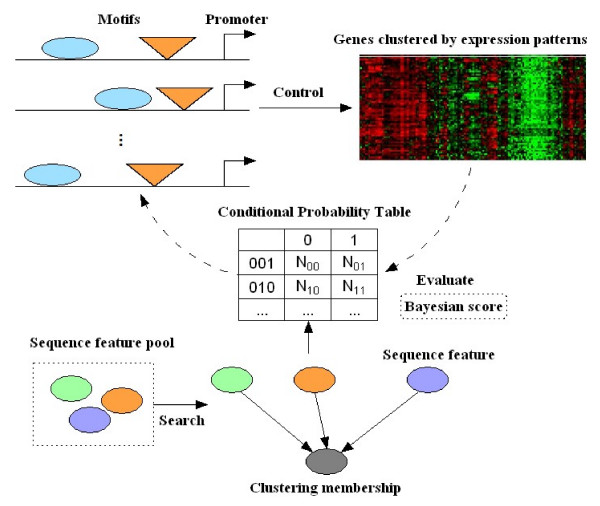
Searching for grammar of combinatorial regulation between transcription factors using a Bayesian network approach.

Uncovering transcriptional grammar in a group of genes exhibiting similar expression patterns may reveal the mechanisms of combinatorial regulation of transcription factors. We adopted a Bayesian network to model the non-linear regulatory relationship between sequence features and gene expression (Fig. 1). The structure of the Bayesian network represents the grammar (regulatory rules) of cis-regulation. Our aim is to maximize the posterior probability of the network structure given the data, i.e. Bayesian score of Eq.(1) (see Methods).

Because the number of sequence features grows exponentially with the number of candidate motifs, searching a set of optimal sequence features is not trivial. We employed a Gibbs sampler enhanced global search strategy to tackle this problem (see Methods for details). Six sequence features as defined in [16] were considered: presence of a motif, distance from transcription start site (TSS), spacing between two motifs, orientation of a motif, presence of a second copy of a motif and order between two motifs. GBNet can therefore be considered as a generalization of sequence motif finding: instead of searching for enriched consensus motifs, enriched combinations of motifs satisfying a specific constraint is being searched.

### Validation of GBNet on simulated data

We first validated the performance of GBNet using simulated data. To keep the sequences as natural as possible, we took the sequences from the 114 promoters in the fourth yeast cluster of [16]. We then implanted a spacing constraint between two yeast motifs, distance between PAC and RRPE motifs less than 40 bp, in a portion of genes ranging from 40% to 80% with an interval of 10%. The original instances of PAC and RRPE were removed. These simulated sequences and the weight matrices of the 666 yeast motifs taken from [16] were input to GBNet for identification of enriched sequence constraints between these motifs. We used the same 1789 background sequences as in [16].

GBNet successfully learned the implemented spacing rule in all the five simulated datasets but BBNet learned none of them (Table 1). For example, when 40% of the genes contained the spacing rule, the presence of single motif PAC and RRPE were ranked 2nd and 5th, respectively, among all motifs under consideration. BBNet only learned the presence of PAC while GBNet still found the spacing constraint between the PAC and RRPE motifs. In all the five datasets, the rules found by GBNet gave better Bayesian scores than those found by BBNet (Table 1), which suggests better fitting to the data. It is important to point out that the Bayesian networks learned by GBNet were not necessarily more complex than those learned by BBNet. On the datasets that 60% and 70% of the genes contained the spacing rule, GBNet even gave Bayesian networks with less number of rules than BBNet (Table 1). Our analysis showed that GBNet outperforms BBNet in search of the best Bayesian network structure even for such a simple simulated data with only one sequence constraint implemented.

**Table 1:** Sequence constraints learned by BBNet and GBNet in the five simulated datasets

Perc^a^	Rank^b^	BBNet		GBNet	
		
		BS	Rules	BS	Rules
0.4	2,5	-130.49	1. Distance to TSS of M604:180 2. Presence of PAC 3. Presence of M599	-123.48	1. Distance to TSS of M604:140 2. **Distance between RRPE and PAC:40** 3. Distance to TSS of M599:160 4. Presence of M593
0.5	1,3	-120.03	1. Presence of PAC 2. Distance to TSS of M604:180 3. Presence of M599	-109.28	1. Distance to TSS of M604:200 2. **Distance between RRPE and PAC:40** 3. Distance to TSS of M599:480
0.6	1,3	-114.19	1. Presence of PAC 2. Distance to TSS of M604:180 3. Presence of RRPE 4. Presence of M599	-102.11	1. **Distance between PAC and RRPE:40** 2. Distance to TSS of M604:140 3. Distance between M604 and M599:500
0.7	1,2	-102.68	1. Presence of PAC 2. Presence of RRPE 3. Distance to TSS of M604:140	-91.18	1. **Distance between PAC and RRPE:40** 2. Distance to TSS of M604:140
0.8	1,2	-85.85	1. Presence of PAC 2. Presence of RRPE 3. Distance to TSS of M604:140	-70.1268	1. **Distance between PAC and RRPE:40** 2. Distance between M604 and M599:340 3. Distance to TSS of M604:140

The MXXX are AlignACE motif matrices taken from [16].

^a^The percentage of sequences satisfying the spacing rule between PAC and RRPE motifs ranges from 0.4 to 0.8. ^b^The single motif ranks for PAC and RRPE in each dataset are also shown: the first is PAC and the second is RRPE.

### Validation of GBNet on the PAC and RRPE example

We then validated the performance of GBNet using a real dataset. We took the fourth yeast cluster of [16] in which Beer and Tavazoie found two regulatory rules for PAC and RRPE: 1. PAC (M600) is within 140 bp of ATG; 2. RRPE (M602) is within 240 bp of ATG. When both rules are satisfied, the genes containing the two motifs showed highly correlated expression patterns across a variety of conditions. When neither of these rules was satisfied, the gene expression patterns were indistinguishable from the background [16].

In [16], the above two rules were learned from the bootstrap samples but not directly from the original sequences in the fourth yeast cluster. We generated numerous sets of 10 bootstrap samples. The two rules could be simultaneously identified in only 1 to 3 out of 10 bootstrap samples by BBNet and they were not necessarily the most abundant rules learned by BBNet from these samples. Different from Beer and Tavazoie's goal to predict gene expression from sequence, we aim to identify sequence constraints between cooperative TF binding motifs from a group of genes with coherent expression patterns. Therefore, bootstrap is not an option for our purpose. When we applied BBNet and GBNet to the original sequences in the fourth yeast cluster, BBNet correctly found the first rule of PAC but only the presence of RRPE instead of the distance constraint (Table S1); In contrast, GBNet successfully found both rules.

**Figure 2:**
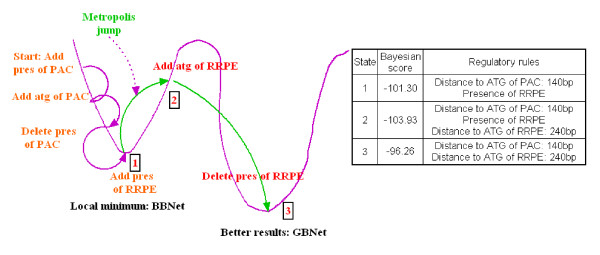
**An example of the Bayesian network learning procedure in BBNet and GBNet.** The sequences were taken from the fourth yeast cluster in [16]. The magenta line represents the landscape of the Bayesian score (absolute value). The learning steps involving motifs other than PAC and RRPE were omitted for the illustration purpose. The parent nodes of the regulator rules learned in the three key steps are shown on the right.

To illustrate why the GBNet could but BBNet could not find the two rules, we examined each step of the Bayesian network structure learning (Fig. 2). When the searching reached a local optimum (state 1 in Fig. 2) with a Bayesian score of -101.3, the network contained two parent nodes (Fig. 2): "distance to ATG of PAC" and "presence of RRPE". If the "distance to ATG of RRPE" node was added, the Bayesian score would decrease. Therefore, the greedy search in BBNet stopped and did not add this rule. The searching was thus trapped in the local optimum. In contrast, a Metropolis jump was tried in GBNet with an accepting probability calculated based on the difference of the Bayesian scores before and after the jump (see Methods): the closer the two Bayesian scores, the more likely a jump got accepted. To further enhance the sampling power, simulated annealing was also employed in GBNet and multiple iterations were executed until the model was converged at a specific temperature. As a result of this searching strategy, the "distance to ATG of RRPE" rule was added by GBNet even though the Bayesian score became worse (state 2 in Fig. 2): -103.93 versus -101.3. Next, the Bayesian score was improved to -96.26 by removing the "presence of RRPE" node (state 3 in Fig. 2). The two correct rules were thus found and being kept to the end of the searching. This example illustrates the advantages of the searching strategy implemented in GBNet to avoid being trapped in the local optimum compared with the greedy search algorithm in BBNet.

### Applying GBNet to the 49 yeast clusters

The above analyses suggested that GBNet can find the rules of combinatorial regulation between TFs. To have a large scale comparison between GBNet and BBNet, we then applied them to the 49 yeast clusters of 2770 genes in [16] (Table S1). We compared GBNet and BBNet on the following aspects using the original data without bootstrap sampling.

#### GBNet fits better models to the data than BBNet

A Bayesian score reflects how well a model fits to the data. The rules learned by GBNet gave better Bayesian scores in 47 clusters than those learned by BBNet (Table S1 in the Additional file 1). The sum of Bayesian scores for all 49 clusters is -4394.3 for BBNet and -4306.6 for GBNet. On average, GBNet achieved a better Bayesian score ~1.8/cluster than BBNet. Again, the Bayesian networks learned by GBNet are not more complex than those learned by BBNet. This can be seen by the average number of rules per cluster: 2.1 for BBNet vs. 2.3 for GBNet.

#### GBNet finds more biologically interesting rules

**Figure 3:**
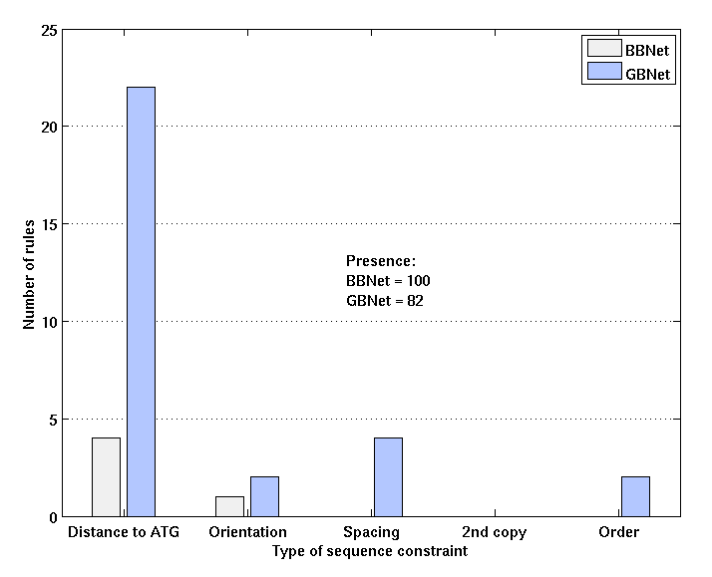
The number of different types of regulatory rules learned by BBNet and GBNet.

From the 49 yeast clusters, BBNet and GBNet learned 105 and 112 regulatory rules in total, respectively. Consistent with the observation in [16], most (100 or 95%) of the regulatory rules learned by BBNet were simply "presence of a motif", which could also be learned by any motif finding algorithm. Because the searching started with "presence of a motif" and BBNet is easy to get trapped in local optima, it is not surprising that other types of sequence constraints were underrepresented. Although presence of a motif is still the majority of the rules learned by GBNet, the percentage is only 73% (82/112) and the portion of other types of constraints was significantly increased (Fig. 3). Finding rules other than presence distinguishes GBNet from other motif finding algorithms. This feature is particularly important in studying combinatorial regulation in higher organisms such as human.

#### GBNet searches more thoroughly in the rule space

**Figure 4:**
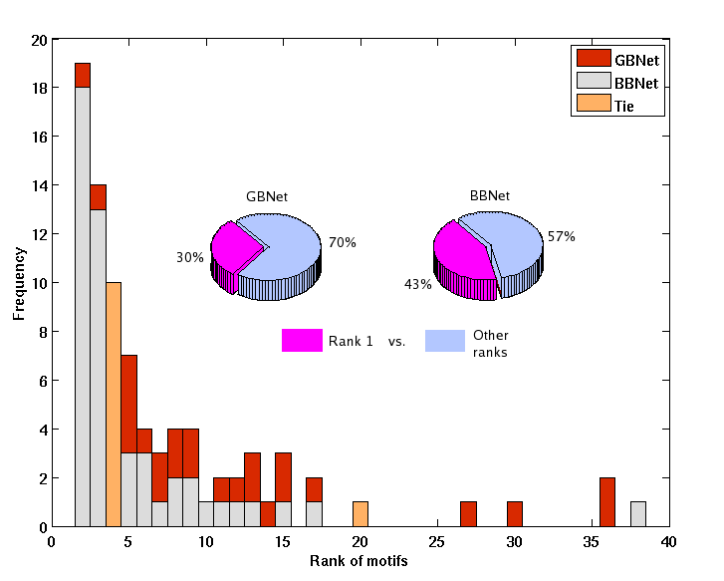
Distribution of motif ranks in BBNet and GBNet. Ties are in orange.

To further demonstrate that GBNet is less prone to get trapped in local optima, we examined the ranks of single motifs that appear in the rules learned by GBNet and BBNet. In search for the optimal Bayesian network, all motifs under consideration were first sorted in the descending order by their individual Bayesian scores, which reflect how well an individual motif can explain the data. The motifs were then added to the Bayesian network in this order to expedite the convergence of searching. Therefore, it is not unexpected to see that a large portion (43%) of motifs present in the rules learned by BBNet had the highest individual ranks (Fig. 4). As a comparison, GBNet found rules that involved motifs giving lower Bayesian score if considered individually (lower individual ranks) but higher (better) Bayesian score if considered together with satisfying specific sequence constraints (Fig. 4).

### GBNet searches much more efficiently in the rule space than enumeration

An alternative to developing models like GBNet for learning sequence constraints is enumerating all possible rules and selecting the best scored ones, using either Bayesian score or mutual information like in FIRE. However, the possible number of combinations of rules is so large to make this straightforward approach computationally prohibited. For example, learning spacing constraints among 50 candidate motifs in yeast, one needs to consider 19 function depths (0.05–0.95 with an interval of 0.05) for each motif and 30 possible distances between two motifs (20–600 bps with an interval of 20 bps). In total, (50 × 49/2) × 19 × 19 × 30 = 13,266,750 statistical tests have to be computed for learning the single spacing constraint. For a distance constraint from TSS (positional bias), 50 × 19 × 30 = 28500 tests need to be performed. Therefore, when consider the combination of the above two rules (spacing constraint and positional bias), there are total 13,266,750*28500 = 3.78*10^11 ^possibilities, which makes enumeration infeasible. As a comparison, on average, GBNet calculated 570,000 times of Bayesian scores per cluster that considered combinations of all six types of constraints. The running time of GBNet per cluster was 3 to 4 hours on a desktop computer with a 1.8 GHz CPU, which means enumeration would take >1.99*10^6 ^to 2.65*10^6 ^hours for only considering the two types of rules mentioned above per cluster. The efficiency difference between GBNet and enumeration becomes more significant when more candidate motifs are considered for learning sequence constrains. This is because GBNet scales better than linearly with the number of candidate motifs (Table S2). As a comparison, the computational cost of enumeration is polynomial to the number of candidate motifs when considering one rule or exponential when considering combination of rules.

### Dissecting transcriptional regulatory rules of a human transcription factor YY1

Combinatorial regulation is much more prevalent in higher organisms than in yeast. To demonstrate the usefulness of GBNet, we applied it to studying transcriptional regulation by a human transcription factor (TF) called YY1, which plays essential roles during development [18-20]. For the purpose of comparison, we also analyzed this human dataset using BBNet. Our previous study showed that YY1 mainly binds to the 1.5 kbp regions around the transcription start site [21]. Therefore, we focus on searching for sequence constraints between YY1 and its cofactors in the proximal promoters.

#### Identifying YY1 target genes and clustering of gene expression profiles

ChIP-chip analysis of YY1 binding has been conducted using a whole-genome promoter array in human HeLa cells [21]. We used a Gibbs sampler based computational algorithm, called **GI**bbs sampler for finding **T**ranscription factor **TAR**get genes (GITTAR) [21] that integrates sequence motif and ChIP-chip binding information to identify a set of confident YY1 target genes. The intuition behind the GITTAR algorithm is the same as that of MODEM [22], namely genes containing the YY1 motif and showing significant ChIP-chip ratio are likely to be YY1 targets. GITTAR identified 968 such genes and the average of their log2 ratios is 2.47 ± 0.70, which significantly deviates from the background (0.23 ± 0.65). A 12-bp long motif defined by GITTAR (Fig. S1 in the Additional file 2) was used in the following analyses.

**Figure 5:**
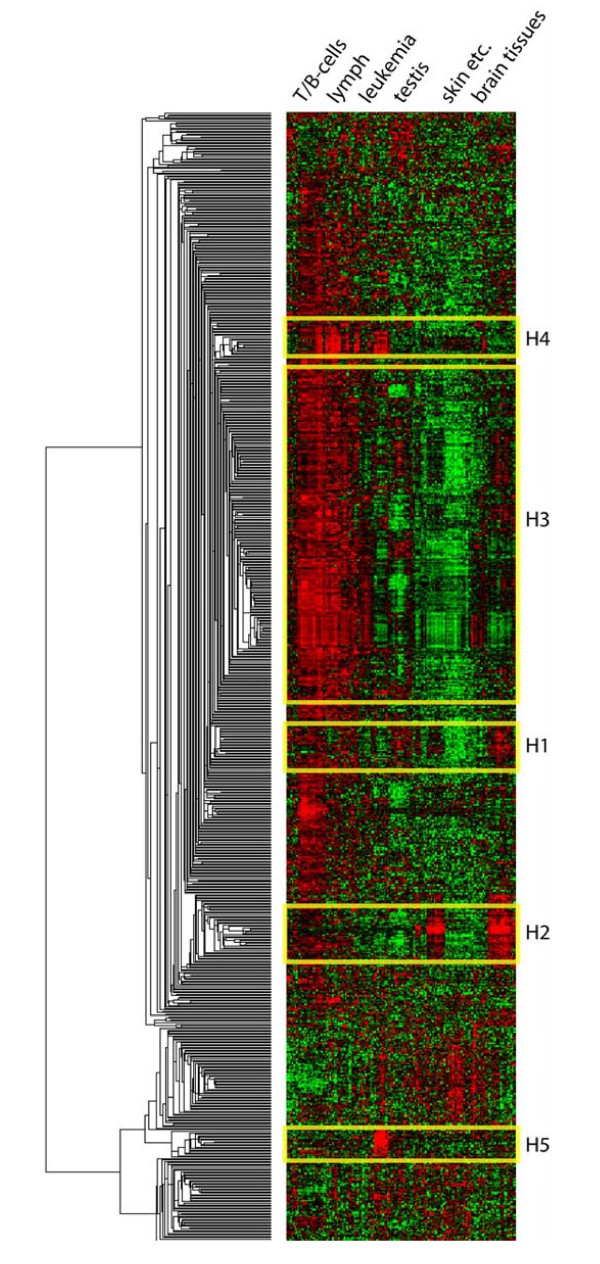
Heatmap of YY1 target gene expression patterns.

Because YY1 can cooperate with various TFs, we used gene expression profiles to define these co-regulated subgroups of the YY1 target genes. Su et al. [23] performed microarray experiments in 79 human tissues and 782 YY1 target genes identified by GITTAR were probed in their arrays. We found five clusters among these YY1 target genes using hierarchical clustering algorithm [24] (Fig. 5). Cluster H1 to H4 were selected based on a correlation cutoff of 0.60 and a cluster size cutoff of 10 genes. Cluster H5 was manually selected because its members were significantly up-regulated and tightly correlated in testis tissues (correlation = 0.64) despite the average pairwise correlation over all the 79 tissues was only 0.33. Cluster H5 represents tissue-specific expression of YY1 targets and it is interesting to examine the underlying mechanism of transcriptional regulation.

#### Finding enriched motifs in each cluster

To search for potential YY1 co-factors, we collected 505 motifs of human TFs from the TRANSFAC database (Version 10.2) [25] and examined their enrichment in each of the five clusters against the genes outside of the cluster but included in the YY1 ChIP-chip study using Fisher's exact test [26] based on hypergeometric distribution (see Methods). In addition, we also conducted *de novo *motif finding using BioProspector [27]. It is not surprising that the YY1 motif was always ranked on the top in Fisher's test. Numerous motifs like E2Fs, CREB, ELK1 and NFY were also significantly enriched. In total, a list of 74 motifs was compiled (Table S3) as the candidate motifs for further analysis of combinatorial rules by GBNet and BBNet.

#### Learning combinatorial regulation between YY1 and its co-factors

**Figure 6:**
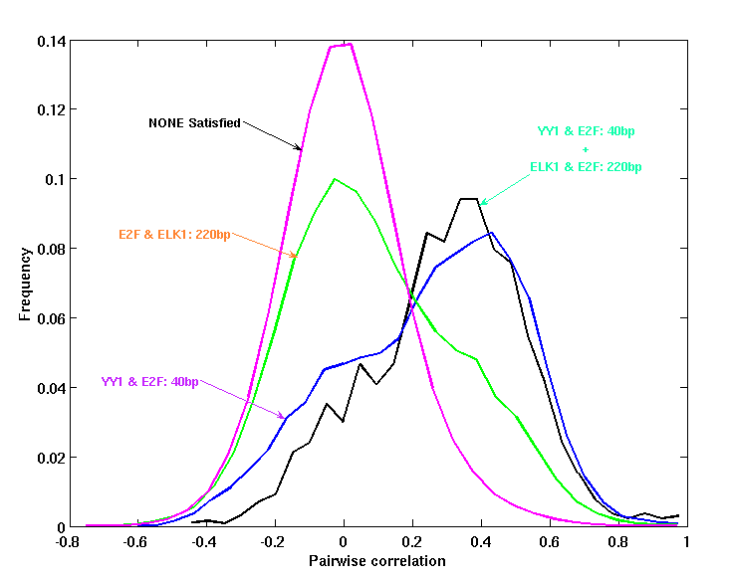
Gene expression pairwise correlation distribution for target genes of the two spacing constraints found by GBNet on cluster H3.

The regulatory rules learned by GBNet and BBNet for all five clusters along with their P-values and Bayesian scores are listed in Table 2. Consistent with the observation in the simulated data and the yeast clusters, GBNet found sequence constraints between cooperative TFs in every cluster while BBNet only learned presence of motifs that can also be found by other means. The GBNet rules also achieved higher Bayesian scores than the BBNet presence rules, which suggest better fitting to the data. Again, the Bayesian networks learned by GBNet are not more complex than those learned by BBNet. In cluster H3, GBNet gave a Bayesian network with one less rule than BBNet but its Bayesian score is 10.0 higher. Two spacing constraints were found on H3: YY1-E2F and E2F-ELK1. We examined the gene expression pairwise correlation (PC) of the target genes of the two spacing constraints. While the E2F-ELK1 pair only marginally raises the PC, the YY1-E2F pair significantly improves the PC compared with background (Fig. 6). This shows the YY1-E2F pair is much more specific than the E2F-ELK1 pair in regulating transcriptional levels of their target genes. Finally, combining the two spacing constraints gives the optimal PC (Fig. 6).

**Table 2:** Sequence constraints learned by BBNet and GBNet in the five human YY1 clusters. The functional depth for each motif is in parentheses.

Cluster	BBNet		GBNet	
	
	Rules, P-value	Bayesian Score	Rules, P-value	Bayesian Score
H1	Presence of YY1 (0.02), 4.05E-12	-18.14	Distance between ETS (0.01) and YY1 (0.02):120 bp, 5.32E-13	-17.23
H2	Presence of YY1 (0.01), 5.43E-10	-22.64	Distance between WT1 (0.02) and YY1 (0.01):40 bp, 1.09E-10	-21.92
H3	Presence of YY1 (0.01), 3.94E-114 Presence of E2F (0.2), 1.54E-33 Presence of ELK1 (0.04), 1.13E-20	-161.70	Distance between YY1 (0.01) and E2F (0.01): 40 bp, 1.67E-121 Distance between ELK1 (0.04) and E2F (0.01): 220 bp, 7.64E-26	-151.71
H4	Presence of YY1 (0.03), 8.64E-6	-20.56	Distance between YY1 (0.01) and E2F1 (0.1): 520 bp, 8.82E-9	-17.71
H5	Presence of YY1 (0.02), 1.90E-6	-21.39	Distance between YY1 (0.02) and ELK1 (0.01):160 bp, 9.79E-8	-19.98

#### Literature evidence to support the rules learned by GBNet

Most of the rules learned by GBNet were supported by literature: YY1 and ETS family proteins have been shown to form a complex that helps to maintain the normal function of human immune system [28]; Both YY1 and ETS are required for the transcriptional regulation of a variety of cellular processes such as adipocyte differentiation [29]; YY1 has been shown to physically interact with E2F family proteins for the specificity of E2F function [30]; Synergistic cooperation has been observed between YY1 and E2F1 for the transcriptional activity of p73, through a mechanism involving a physical interaction [31]; Two independent groups have verified that YY1 and ELK1 co-ordinate the expression of the SOD gene [32,33]. In addition, GBNet also identified two new cooperative pairs: YY1-WT1 and E2F-ELK1. It is worth pointing out that GBNet specifies how the above TFs cooperative with each other and provides specific guidance for experimental validation.

#### Independent E2F ChIP-chip experiments support the YY1-E2F distance constraint

A direct evidence to support the sequence constraints found by GBNet came from a recent study on the binding of E2F family members. Farnham and colleagues recently conducted ChIP-chip experiments on E2F family members, E2F1, E2F4 and E2F6, using the same promoter array that we used for our YY1 study (see Methods and [34]). They showed that E2F family members mainly bind to promoter regions within 2 kb of transcription start site (TSS) in HeLa cells and their bindings are interchangeable [34]. Among the 2815 human genes that are targets of any of the three E2F family members, 496 are in common with the 968 GITTAR YY1 targets (P-value < 2.5e-167 based on hypergeometric distribution).

**Figure 7:**
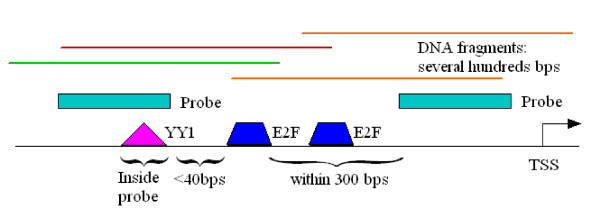
**YY1 and E2F pairs predicted by GBNet were confirmed by ChIP-chip experiments.** 79% of the 170 YY1-E2F pairs constrained by the distance were found to have probes with significant binding ratio change (more than 2-fold) within 300 bps.

To confirm the distance constraint between YY1 and E2F family members, we examined how many of YY1 and E2F sites that satisfy the distance constraint (within 40 bp) were supported by the E2F ChIP-chip experiments (Fig. 7). Because the probes in the promoter array were not uniformly distributed in each promoter, a predicted E2F site by GBNet may fall into a gap between probes. In addition, the sonicated DNA segments in ChIP-chip experiments had a length of hundreds of base pairs. Therefore, if a predicted E2F site is within a short distance from a probe with significant binding ratio, it is likely the E2F proteins bind to the predicted site. Among the 170 YY1-E2F motif pairs predicted by GBNet in the cluster H3 genes, we found that 79% of them were close to a probe (within 300 bp) with significant binding ratio of more than 2-fold (Table S4) (see Additional file 2 for more details). As a control, 104 genes which contain an YY1 site but do not satisfy the YY1-E2F spacing constraint were selected from the genome. Among these control genes, only 20% contain a probe (within 300 bp) with significant binding ratio of more than 2-fold. The statistical significance (p-value = 1.4e-22) was evaluated by Fisher's exact test between the two groups. This suggests that most of the predicted E2F sites by GBNet were bound by E2F proteins and the majority of the YY1-E2F distance constraints identified by GBNet were thus supported by the E2F ChIP-chip experiments.

## Discussion and conclusion

Combinatorial regulation of transcription factors is critical in gene expression control particularly in higher organisms. For the purpose of reconstructing transcription regulatory network, understanding the molecular mechanisms and deciphering the grammar of combinatorial regulation are the natural steps after finding the binding sites of TFs [3]. Identification of cooperative TFs and learning sequence constraints between their motifs can provide great insights into building mechanistic and quantitative models of transcriptional regulation [22].

We have developed a Bayesian network approach to find regulatory rules enriched in a foreground sequences, for example the promoters of a set of co-regulated genes, compared with the background sequences. This method can be applied to any genome as we showed its success in yeast and human here. We designed a powerful searching strategy in Bayesian network structure learning by employing Gibbs sampling and simulated annealing. Compared with the exhaustive enumeration, GBNet can find the optimal rules much more efficiently. The more candidate motifs under consideration, the more save of computational cost GBNet would achieve over enumeration. Given the improved searching strategy, it is not surprising that GBNet outperforms BBNet that employs greedy search for optimal network structure in all the datasets we have tested, including simulated, yeast and human data.

In the present study, we were focused on the six sequence constraints using in [16] and analyzing combinatorial regulations in proximal promoters. Obviously, there exist other sequence constraints, particularly those for the interactions between distal enhancers and promoters and those related to other regulatory elements such as silencers. New approaches are emerging to define all the regulatory elements, for example, using chromatin modification patterns [35] or protein-DNA interaction data to predict enhancers [36]. The accumulation of such knowledge can facilitate GBNet to learn rules that involve other regulatory elements or long-range regulatory interactions such as the looping interaction between distal enhancers and promoters. It is straightforward to search for other types of rules by GBNet without significantly increasing the computational cost.

## Methods

### GBNet

The model fitness of the Bayesian network can be evaluated by Equation 1 in [16], which is the posterior probability of the network structure given data [16]. To minimize the round-off errors, we use the log-value of this posterior probability to define the Bayesian score in this paper:

(1) $$\log_{10}(P(N_{s}|D))=-N_{p}\log_{10}(K)+\sum_{j=1}^{q}\log_{10}\frac{\Gamma (a_{j})}{\Gamma (a_{j}+N_{j})}\sum_{k=0}^{r}\log_{10}\frac{\Gamma (a_{jk}+N_{jk})}{\Gamma (a_{jk})}$$

where *N*_*s *_is network structure, *D *is data, Γ (·) is the gamma function, *N*_*p *_is the number of parent nodes, log_10_(*K*) is a network parameter (see below), *q *is the number of possible parent states, *r *+ 1 is the number of possible child states, *a*_*j *_= Σ *a*_*jk*_, *N*_*j *_= Σ *N*_*jk*_, *N*_*jk *_is the number of samples for child state *k *when parent state is *j*, *a*_*jk *_is prior count. In BBNet, a greedy search algorithm was employed to search for the best network structure: a search stopped when adding a new parent node (sequence constraint) could not further improve the Bayesian score. This procedure is prone to get trapped in local optimum. To improve the searching efficiency, we implemented a Metropolis jumping in GBNet each time when a parent node (sequence constraint) was added or the functional depth of a motif was updated. In addition, simulated annealing was also exploited to search for the global optimum (see Fig. S2 for a comparison between the two search algorithms). A change to the Bayesian network structure was accepted by a probability of $\min\left(1,\;{\left(\frac{P({N^{\prime }}_{s}|D)}{P(N_{s}|D)}\right)}^{\frac{1}{T}}\right)$, where ${N^{\prime }}_{s}$ and *N*_*s *_are network structures after and before the change and *T *is the temperature. In simulated annealing, *T *was decreased exponentially as *T *← α^n^*T*, where *n *is the number of iterations and α is the rate of change. The searching procedure stopped when there was no change detected at a specific temperature after a number of attempts or a sufficient number of temperature changes had been made. In this work, the initial temperature was set to 5.0 and α = 0.9 for both yeast and human data. The simulation moved to the next temperature if either the number of iterations reached 20 or the number of structure changes reached 500. The maximum number of temperature changes was set to 20. In our tests, GBNet was always able to find the optimum using these parameters.

The background sequences were selected as the following. For the yeast data, the same background as Beer and Tavazoie (2004) was used for a fair comparison. For the human YY1 data, the number of background sequences was set to five times of the size of the cluster under consideration. This background size was heuristically determined to achieve a balance between discrimination and statistical significance. All genes were ranked according to their correlation to the mean expression profile of the cluster in the ascending order and the least correlated or the most anti-correlated genes were selected as the background. The structural parameter, log_10 _*K *in Eq.(1), helps avoid overfitting in learning the Bayesian network structure by penalizing the complex network structures [16]. The value of log_10 _*K *in [16] was used for the yeast data and a heuristic value of 5.0 was chosen for the human YY1 data.

### Finding enriched co-factors using Fisher's exact test

Fisher's exact test can evaluate the significance of the association between two variables [26]. The test is implemented through the use of a 2 × 2 contingency table. When testing the significance of motif enrichment for a cluster, we designed the contingency table 3 as follows

**Table 3:** Contingency table

	Within-cluster	Outside-cluster	Total
Match motif	*a*	*b*	*a *+ *b*
Non-match	*c*	*d*	*c *+ *d*
Total	*a *+ *c*	*b *+ *d*	*n*

where *a*, *b*, *c*, *d *are the numbers of genes in each category; *n *= *a*+*b*+*c*+*d *is the total number of genes under consideration. The same criterion as described above was used to select candidate motifs.

### The YY1 ChIP-chip data

The YY1 ChIP-chip data was obtained from our previous study [21]. Briefly, the whole-genome promoter array was designed and synthesized by Nimblegen. 24134 promoters from human genome build 35 (HGS17) were represented on the array. 1500 bp sequence of each promoter (1300 bp upstream and 200 bp downstream of TSS) is covered by 15 oligo probes of 50 bps long. Two replicates of experiments were conducted in HeLa cells. Data were collected and processed as described previously [37,38].

## Authors' contributions

LS and WW conceived the experiments and wrote the manuscript. LS carried out the data analysis and implemented the software. JL participated in the data analysis. All authors read and approved the final manuscript.

## Supplementary Material

Additional file 1 **Microsoft Excel file containing results of Bayesian score, rules, etc. of 49 yeast clusters from both BBNet and GBNet.** Click here for file

Additional file 2 **Microsoft Word file containing supplemental information of the main article, Fig. S1–S2 and Table S2–S4.** Click here for file
