# Supplementary material for: GBNet: Deciphering regulatory rules in the co-regulated genes using a Gibbs sampler enhanced Bayesian network approach
Source: BMC Bioinformatics. 2008 Sep 24;9:395. doi: 10.1186/1471-2105-9-395 (PMC2571992; doi:10.1186/1471-2105-9-395)
Supplement: Additional file 2 — Microsoft Word file containing supplemental information of the main article, Fig. S1–S2 and Table S2–S4. [file 1471-2105-9-395-S2.doc]

**Validation of YY1-E2Fs spacing constraint by independent ChIP-chip experiments**

The E2F ChIP-chip experiments using promoter array in human HeLa cells were taken from Xu et al.(Xu et al. 2007). Each array experiment contained two replicates and the log2 ratios were averaged. Same as the analysis by Xu et al., the log2 ChIP-chip ratio for each promoter was calculated based on the median of the top 11 of the 15 probes in the promoter region. A gene was considered as an E2F target if at least 2-fold ratio was detected in any of the E2F1, E2F4 and E2F6 arrays. Because the YY1-E2F sites found by GBNet were not necessarily covered by any of the 15 probes in a promoter, we first identified the closest probes to these YY1 and E2F sites and these probes should be within 300bp of the predicted sites. Because the DNA segments in ChIP-chip analysis are usually several hundreds base pairs long, a probe that is 300bp away from a TF binding site should still show significant ratio when the TF binds. If both of the YY1 and E2F probes were associated with a ChIP-chip ratio of 2 folds, we considered the distance constraint between the YY1-E2F pair being confirmed. YY1 site is specific and most promoters only contain one copy of YY1 site. Therefore, we considered all YY1 sites in each promoter. When a promoter contains multiple copies of the E2F sites that satisfy the distance constraint (within 40bp) to a YY1 site, the strongest E2F site was used to select the closest probe in the promoter array. The above analysis confirmed 79% of the YY1-E2F pairs that satisfy the distance constraint in the human HeLa cells.


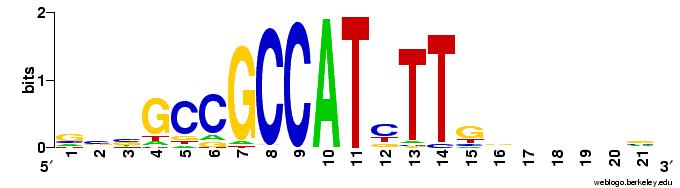


Fig. S1 Sequence logo of YY1 motif in human. Only the most informative 12 positions (4-15) were used in finding regulatory rules of YY1 and its cofactors by GBNet and BBNet.


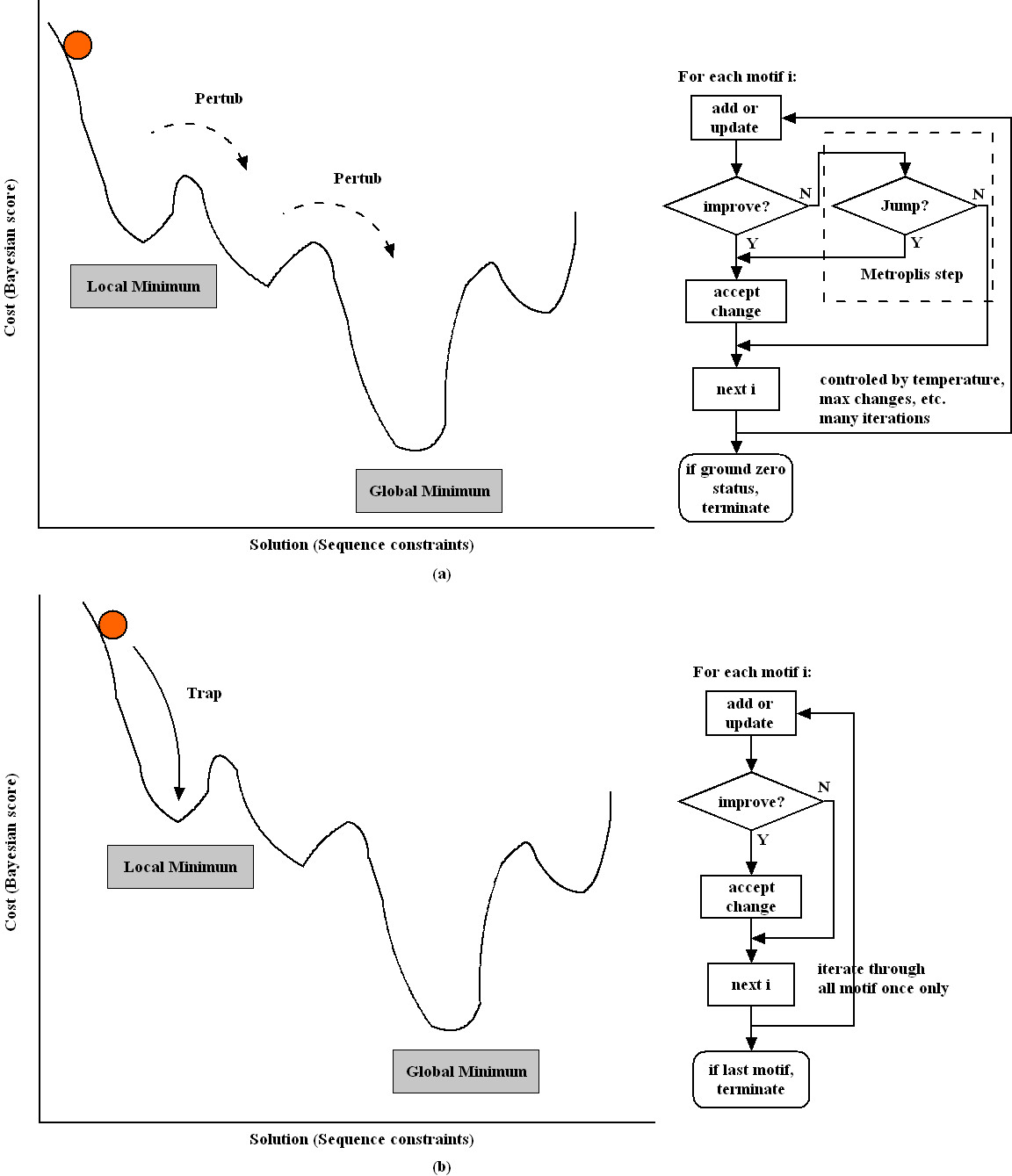


Fig. S2 The Bayesian network learning strategies in (a) GBNet and (b) BBNet.

Table S2 Number of tests done by GBNet using different number of top motifs in the yeast cluster 4 in (Beer and Tavazoie 2004).

| Top motifs | Tests |
| --- | --- |
| 25 | 439,850 |
| 50 | 709,935 |
| 100 | 1,113,077 |
| All(666) | 5,180,483 |

Table S3 Enriched TRANSFAC motifs in the five human YY1 clusters. Enriched motifs were selected based on P-value < 0.05 and the number of matches (proportion) ≥ 25% of the cluster size.

| Cluster | Motif | Proportion | P-value |
| --- | --- | --- | --- |
| H1 | YY1_Q6  YY1_Q6_02  NFY_Q6  LEF1TCF1_Q4  FREAC7_01  P53_02  E2F1_Q3  P53_DECAMER_Q2  SREBP_Q3  MYB_Q6  MYB_Q3  E2F_Q2  CREB_Q3  PAX4_01 | 0.95  0.84  0.26  0.47  0.37  0.32  0.84  0.37  0.42  0.63  0.63  0.95  0.79  0.42 | 8.90E-12  1.28E-07  1.22E-03  1.26E-02  1.41E-02  1.77E-02  1.84E-02  2.14E-02  2.50E-02  3.55E-02  3.55E-02  3.60E-02  3.74E-02  3.79E-02 |
| H2 | YY1_Q6  YY1_Q6_02  CREBATF_Q6  SP1_Q6  AHR_Q5  MAZ_Q6  E2A_Q2  SREBP_Q3  CREB_Q4_01  MYB_Q5_01  SP1_Q2_01  SP1_Q4_01 | 0.81  0.71  0.29  0.76  0.48  0.57  0.90  0.43  0.43  0.95  0.48  0.48 | 6.97E-09  1.26E-05  1.78E-03  2.91E-03  6.26E-03  1.66E-02  1.91E-02  1.95E-02  3.23E-02  3.93E-02  4.98E-02  4.99E-02 |
| H3 | YY1_Q6  YY1_Q6_02  E2F_Q2  ETF_Q6  E2F1_Q3  CREB_Q3  E2F1_Q4  NFY_Q6_01  AP4_Q5  PEA3_Q6  LBP1_Q6  WT1_Q6  HIC1_02  EN1_01  CETS1P54_01  AHRHIF_Q6  ZF5_B  CREB_Q2_01  MYOD_Q6  E2A_Q6  LFA1_Q6  AHR_Q5  TEF1_Q6  AREB6_01  GATA1_03  NF1_Q6  GATA3_01  RFX_Q6  HOXA4_Q2  AP2_Q6_01  MYB_Q5_01  BRCA_01  AREB6_03  CEBP_Q2_01  PR_Q2  ZF5_01  STAT1_02  PITX2_Q2  AP2ALPHA_01  BACH2_01  TBP_Q6  SP1_Q6  AP1_Q2  CRX_Q4 | 0.80  0.71  0.96  0.97  0.75  0.70  0.29  0.30  0.28  0.60  0.30  0.96  1.00  0.98  0.50  0.68  0.61  0.46  0.31  0.31  0.35  0.29  0.88  0.63  0.86  0.98  0.54  0.34  0.33  0.96  0.83  0.60  0.57  0.65  0.44  0.40  0.42  0.45  0.94  0.30  0.34  0.49  0.67  0.91 | 5.30E-84  8.92E-49  1.18E-19  4.36E-10  2.81E-09  6.62E-07  1.14E-06  4.54E-06  1.47E-05  1.76E-05  2.14E-05  6.68E-05  3.90E-04  7.98E-04  1.23E-03  2.20E-03  2.55E-03  2.65E-03  4.04E-03  4.04E-03  4.49E-03  4.98E-03  5.04E-03  5.66E-03  7.23E-03  8.25E-03  8.47E-03  9.07E-03  1.04E-02  1.15E-02  1.41E-02  1.53E-02  2.03E-02  2.04E-02  2.16E-02  2.17E-02  2.31E-02  2.57E-02  2.95E-02  3.76E-02  3.88E-02  3.94E-02  3.94E-02  4.30E-02 |
| H4 | YY1_Q6  YY1_Q6_02  ELK1_02  E2F1_Q4  E2F1_Q6  E2F_Q4  E2F_Q6  STAT5B_01  AREB6_01  MZF1_02  E2F_Q2  IPF1_Q4_01  STAT5A_01  ETS_Q6  PAX4_01  RBPJK_01  CEBPB_02  SP1_Q6 | 0.81  0.81  0.38  0.50  0.31  0.31  0.31  0.25  0.38  1.00  1.00  0.38  0.38  0.38  0.44  0.31  0.88  0.69 | 4.32E-07  4.60E-06  1.74E-03  1.79E-03  2.24E-03  2.24E-03  2.24E-03  4.46E-03  4.69E-03  9.42E-03  9.76E-03  1.99E-02  2.33E-02  2.69E-02  2.73E-02  3.12E-02  3.90E-02  4.05E-02 |
| H5 | YY1_Q6  YY1_Q6_02  ATF4_Q2  E2F1_Q4  STRA13_01  ELK1_02  CETS1P54_01  MEIS1_01 | 0.76  0.76  0.35  0.41  0.41  0.29  0.65  0.76 | 1.48E-06  1.50E-05  1.23E-02  1.27E-02  1.34E-02  1.34E-02  4.41E-02  4.90E-02 |

Table S4 Validation of YY1-E2F distance constraints. 170 YY1-E2F pairs satisfying the distance constraint (within 40bp) in H3 predicted by GBNet.

| Distance^a^ | Number of genes |
| --- | --- |
| Within the same probe (0) | 44 |
| (0, 300] | 91 |
| (300, ∞) | 17 |
| No co-bound | 18 |

^a^ The largest distance between the closest probes to the YY1 and E2F sites.

**References:**

Beer M, Tavazoie S (2004) Predicting gene expression from sequence. Cell 117: 185-198.

Xu X, Bieda M, Jin VX, Rabinovich A, Oberley MJ et al. (2007) A comprehensive ChIP chip analysis of E2F1, E2F4, and E2F6 in normal and tumor cells reveals interchangeable roles of E2F family members. Genome Res: gr.6783507.
